# Biomedical and life science articles by female researchers spend longer under review

**DOI:** 10.1371/journal.pbio.3003574

**Published:** 2026-01-20

**Authors:** David Alvarez-Ponce, Gabrial Batz, Luis Ramirez Torres

**Affiliations:** Biology Department, University of Nevada, Reno, Nevada, United States of America; University of Bath, UNITED KINGDOM OF GREAT BRITAIN AND NORTHERN IRELAND

## Abstract

Women are underrepresented in academia—especially in STEMM fields, at top institutions, and in senior positions. This is due, at least in part, to the many obstacles that they face compared to their male counterparts. There has been substantial debate as to whether the peer review system is biased against women. Some studies—mostly based on analyses of thousands of Economics research articles—have shown that manuscripts authored by women experience longer peer review times (defined as the time intervened from submission to acceptance) than comparable manuscripts authored by men. Other studies, however, have found no effect of author’s gender on acceptance delays, raising questions about whether the gender gap is specific to certain fields. Biomedical and life scientists produce 36% of the research articles published annually worldwide; therefore, a comprehensive understanding of how women are treated by the peer review system requires a thorough examination of biomedicine and the life sciences. By analyzing all articles indexed in the PubMed database (>36.5 million articles published in >36,000 biomedical and life sciences journals), we show that the median amount of time spent under review is 7.4%–14.6% longer for female-authored articles than for male-authored articles, and that differences remain significant after controlling for several factors. The gender gap is pervasive, affecting most disciplines, regardless of how well women are represented in each discipline; however, the gap is absent or even reversed in some disciplines. We also show that authors based in low-income countries tend to experience longer review times. Our findings contribute to explaining the gender gap in publication rates and representation.

## Abstract 2

Women are underrepresented in academia, especially in STEMM fields, at top institutions, and in senior positions. This study analyzes millions of biomedical and life science articles, revealing that female-authored articles spend longer under review than comparable male-authored articles, across most fields.

## Introduction

Women are underrepresented in academia—especially in STEMM fields, at top institutions, and in senior positions [1–6]. This is largely because women are more likely than men to leave academia, whether voluntarily or involuntarily [7,8]. At least in part, this trend is due to female academics facing additional obstacles throughout their careers compared with their male counterparts. First, women are often subjected to specific societal demands (e.g., a disproportionate amount of childcare and home responsibilities) that can limit the amount of time that they can devote to their careers [9–11]. Second, on average they face higher teaching and service loads than their male counterparts, which limits the amount of time that they can devote to their research [12,13]. Third, on average they receive less recognition for their work: they are more likely to be excluded as co-authors of publications [14] and less likely to obtain research funding [7,15], their work tends to be less visible [16–18] and cited [6,19,20], they are less likely to be invited to present their work at conferences [21] or to submit their manuscripts to journals [4], evaluators tend to evaluate articles less favorably when they think that they have been written by women [22–24], and they are paid less than men (even after controlling for seniority [25]). This may contribute to explain why women on average publish at lower rates [6,26–28] and are less likely to obtain tenure and being promoted [29].

Documenting the different dimensions of the gender gap in academia will help the academic community be cognizant of them and devise mitigation strategies. Academics’ career advancement largely depends on publication records. Thus, it is crucial to understand whether the editorial and peer review systems is biased against female researchers. While female- and male-authored articles seem to be accepted at similar rates [30–36], some studies have shown that female-authored research articles spend significantly longer under review (defined as the time intervening from submission to acceptance) than male-authored ones.

By analyzing 79,189 manuscripts submitted to the 15 journals published by the American Society for Microbiology, Hagan and colleagues [37] found that female-authored articles take on average 1–9 days longer to be accepted, despite them spending a similar amount of time in the society’s peer review system. By analyzing 4,435 articles published in two top Economics research journals, Hengel [38] showed that female-authored articles spend on average 3–6 months longer under review than comparable male-authored articles, a difference that remained significant after controlling for several confounding factors. An analysis of 26,919 articles published in 33 Economics and Finance journals showed that referees spend longer reviewing female-authored articles, that female authors spend longer revising their manuscripts, and that female-authored articles are subjected to more rounds of peer review [39]. An analysis of 44,371 articles published in 255 Turkish national journals showed that average times under review are the lowest for articles authored by a solo male author, low for articles by a solo female author, intermediate for articles by all-male teams, high for articles by mixed-gender teams, and the highest for articles by all-female teams [40]. An analysis of 825 articles published in three top Accounting journals showed that articles with a female corresponding author spend on average longer under review, and that a mismatch in the gender of the corresponding author and the handling editor often results in increased delays [41]. Finally, an analysis of 5,548 articles focusing on Economics showed that female-authored articles spend longer under review, and that the gender gap is smaller for areas in which women are better represented [42].

Other studies, however, have found little or no differences in how long female- and male-authored research articles spend under review. An analysis of 15,147 manuscripts submitted to four top Economics research journals found that authors’ gender has no significant effect on the amount of time taken by editors to make decisions, the amount of time spent by referees to submit their reviews, or the total submission-to-decision time [43]. By analyzing 31,420 articles published in 11 Evolutionary Biology journals, Alvarez-Ponce and Vesper [44] found that female-authored articles experience longer submission-to-acceptance times in some journals; however, most of the differences disappear after controlling for confounding factors.

These conflicting results indicate that whether female researchers tend to experience longer review times is a complex question, whose answer may depend on an array of factors such as the specific field of research. Whether the trend affects most academic fields or only some—such as those in which women are least represented—remains unknown. Combined, biomedicine and the life sciences represent the most active research area worldwide, accounting for 36% of research articles produced annually [45], and 38% of researchers working in these disciplines globally are women [4]. Thus, to gain a comprehensive understanding of how female researchers are treated by the editorial and peer review system, it is essential to understand their experiences across the different biomedical and life science fields.

The primary aim of our study is to determine whether female-authored articles spend longer under review. To test this hypothesis, we analyzed all articles (over 36.5 million articles from over 36,000 academic journals) indexed in the PubMed database, the main biomedicine and life sciences abstract indexing database (https://pubmed.ncbi.nlm.nih.gov/). We found that: (*i*) Even though female representation has dramatically increased in the last decades, women are still underrepresented among authors of biomedical and life sciences papers, especially among corresponding authors. (*ii*) The median time under review was 7.4% longer for articles with a female first author than for articles with a male first author, 12.7% longer for articles with a female corresponding author than for articles with a male corresponding author, 14.6% longer for articles with a female first author and a female corresponding author than for articles with a male first author and a male corresponding author, and 10.0% longer for articles authored by all-female teams than for articles authored by all-male teams. (*iii*) The differences remain significant after controlling for several confounding factors. (*iv*) The trend applies to most biomedical and life science fields (regardless of how well women are represented in each field) and affects authors from most countries. (*v*) Authors based in low-income countries experience review times than are 25.8%–44.3% longer than those for other countries, but the trend affects female and male authors equally.

## Results

### Women are underrepresented in biomedical and life sciences research, especially among corresponding authors

We extracted a total of 36,555,430 abstracts and their associated metadata from the PubMed database (Dataset 1). The corresponding articles were published between 1781 and 2024 in 36,388 biomedical and life sciences academic journals. Combined, these articles had a total of 160,853,266 authors, of which 29,349,577 were inferred to be female based on their first name (for simplicity, we will refer to these authors as “female authors” throughout this manuscript), 53,225,315 were inferred to be male based on their first name (for simplicity, we will refer to these authors as “male authors”), 19,862,715 had unisex names, and 58,415,659 had names of unknown gender or only their initials were available. Thus, out of the 82,574,892 authors whose gender could be inferred, 35.5% were female.

Among the first authors of the 36,555,430 articles indexed in the database, 6,005,519 were female, 9,319,567 were male, 3,536,630 had unisex names, and 17,693,714 had names of unknown gender, only their initials were available, or their names were not listed in PubMed. Thus, out of the 15,325,086 first authors whose gender could be inferred, 39.2% were female.

Out of the 36,555,430 articles indexed in the database, we could identify the corresponding author or authors of 9,283,504. If more than one was identified for any given article, only the last one was retained, since the last corresponding author tends to carry the most supervisory responsibilities [46,47]. Out of these corresponding authors, 2,039,947 were female, 4,221,269 were male, 1,181,439 had unisex names, and 1,840,849 had names of unknown gender or only their initials were available. Thus, out of the 6,261,216 corresponding authors whose gender could be inferred, 32.6% were female.

Out of the 36,388 journals indexed in the database, 11,494 had at least 50 authors (across all the articles published in the journal) whose gender could be inferred (Dataset 1b). Among these journals, the fraction of female authors ranged from 0% to 100%, with a median value of 36.5% (S1 Fig).

### Female representation among authors substantially increased over time

**Fig 1:**
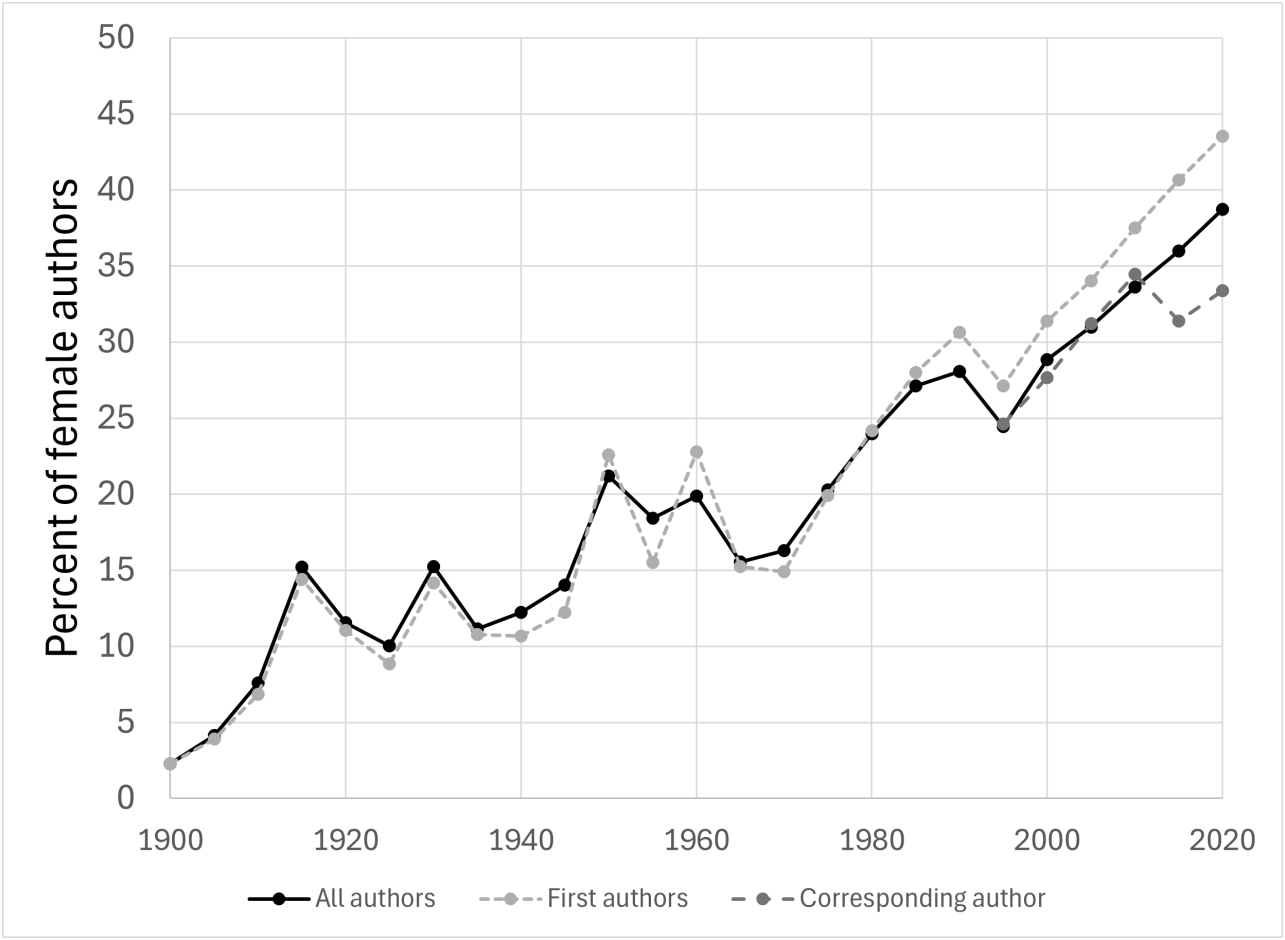
Female representation among the authors of 34,013,183 biomedical and life science articles published between 1900 and 2024. Articles were grouped into quinquennia. For graphing purposes, percentages corresponding to each quinquennium (1900–1904, 1905–1909, etc.) were assigned to the first year of the quinquennium (1900, 1905, etc.). Since corresponding authors were inferred based on availability of email addresses, we only computed the percentage of female corresponding authors since the 1995–1999 quinquennium. This figure is based on Dataset 1 (excluding articles published before 1900). The data underlying this Figure can be found in Zenodo (https://doi.org/10.5281/zenodo.17796183).

To study how female representation changed over time in biomedicine and the life sciences, we grouped articles published between 1900 and 2024 into quinquennia (1900–1904, 1905–1909, etc.) and calculated the percentage of female authors among all authors, among the first authors, and among the corresponding authors of articles within each quinquennium. We observed that female representation dramatically increased over time: women only accounted for 2.3% of the authors of articles published in 1900–1904, while they accounted for 38.7% of the authors of articles published in 2020–2024 (Fig 1). Similarly, the percentage of women among first authors increased from 2.3% in 1900–1904 to 43.5% in 2020–2024, and the percentage of women among corresponding authors increased from 15.4% in 1995–1999 to 33.4% in 2020–2024.

### Female-authored articles tend to spend longer under review

For each article, we computed the time that it spent under review as the time intervened from its submission date to its acceptance date. We removed all articles whose submission and/or acceptance dates were not available, and those that spent less than one day under review. In addition, for each journal we discarded those articles that did not belong to the most common article category for that journal (for most journals, the most common category was “Journal article”) to avoid potential biases due to certain types of articles typically experiencing shorter review times (e.g., “Letter”) or not going through traditional peer review (e.g., “Editorial”). This resulted in 7,758,839 articles published in 8,860 journals (Dataset 2). Out of these articles, 2,562,262 had a female first author, 3,405,821 had a male first author, 975,010 had a female corresponding author, 1,946,469 had a male corresponding author, 757,878 had both a female first author and a female corresponding author, 1,357,835 had both a male first author and a male corresponding author, 650,280 were authored by all-female teams, and 2,146,799 were authored by all-male teams.

**Fig 2:**
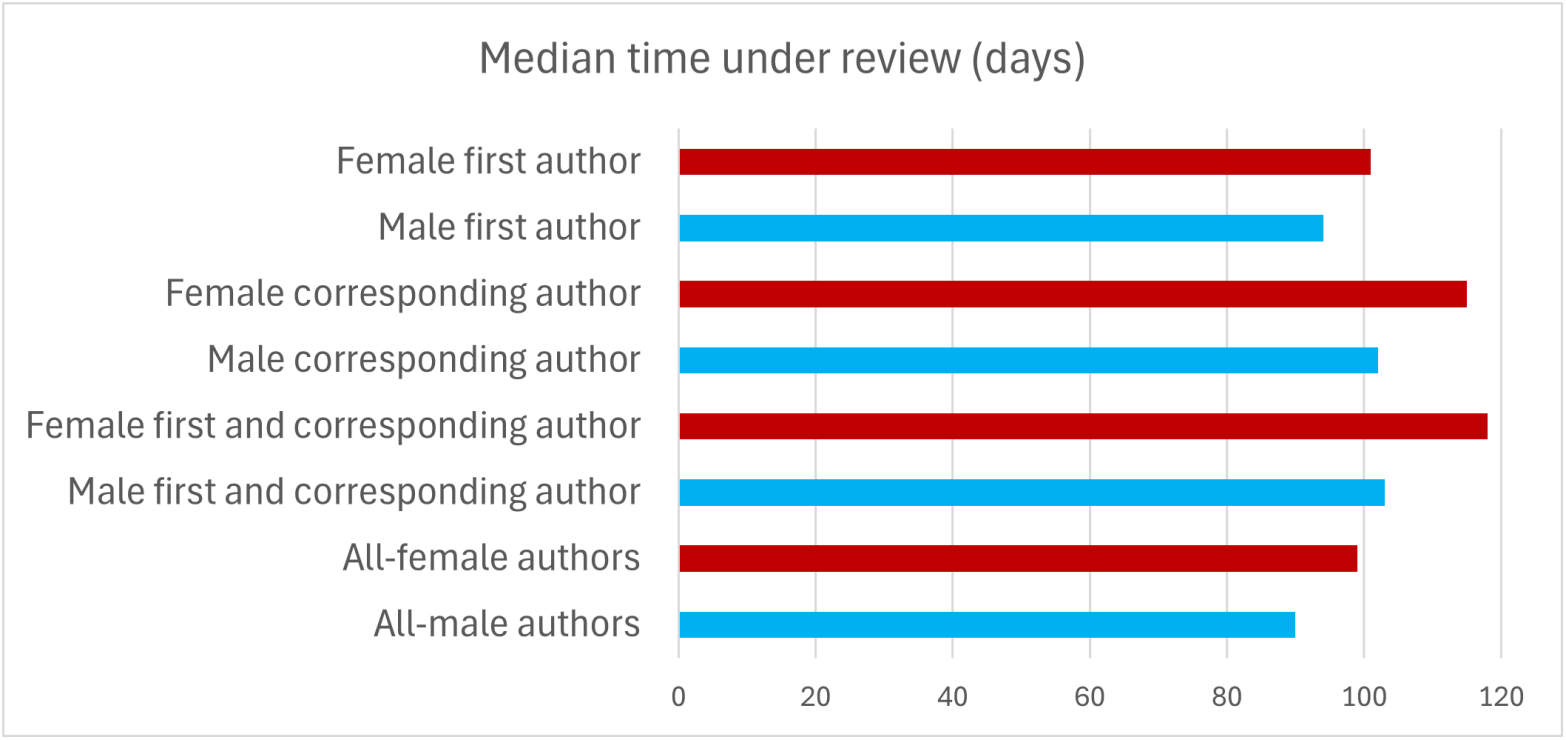
Median time under review (time intervened from submission to acceptance) of articles indexed in PubMed with a female first author (*n* = 2,562,262), a male first author (*n* = 3,405,821), a female corresponding author (*n* = 975,010), a male corresponding author (*n* = 1,946,469), a female first author and a female corresponding author (*n *= 757,878), a male first author and a male corresponding author (*n *= 1,357,835), all-female authors (*n *= 650,280), and all-male authors (*n *= 2,146,799). This graph is based on Dataset 2. The data underlying this Figure can be found in Zenodo (https://doi.org/10.5281/zenodo.17796183).

Articles with a female first author spent significantly longer under review (median: 101 days) than articles with a male first author (median: 94 days; Mann–Whitney’s *U* test, *P* < 10^−290^; Fig 2). Articles with a female corresponding author spent significantly longer under review (median: 115 days) than articles with a male corresponding author (median: 102 days; Mann–Whitney’s *U* test, *P* < 10^−290^; Fig 2). Articles with a female first author and a female corresponding author spent significantly longer under review (median: 118 days) than articles with a male first author and a male corresponding author (median: 103 days; Mann–Whitney’s *U* test, *P* < 10^−290^; Fig 2). Articles authored by all-female teams spent significantly longer under review (median: 99 days) than articles authored by all-male teams (median: 90 days; Mann–Whitney’s *U* test, *P* < 10^−290^; Fig 2).

For each article, we calculated the fraction of female authors as the number of female authors divided by the number of authors whose gender could be inferred (female + male). This fraction exhibited a weak but significantly positive correlation with the amount of time that articles spent under review—i.e., articles with a high fraction of female authors tend to experience longer review times (Spearman’s rank correlation coefficient, *ρ* = 0.049, *P* < 10^−290^).

**Fig 3:**
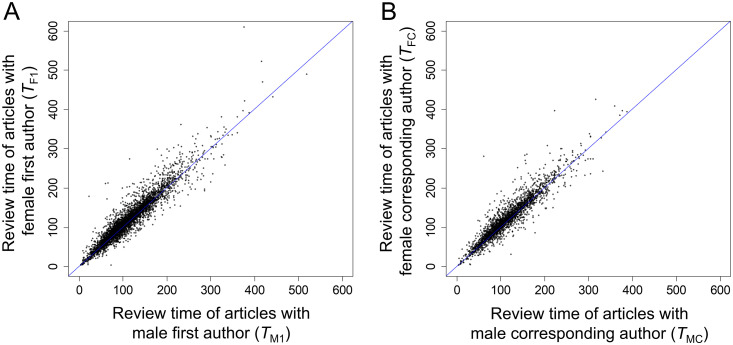
Correlation between the median time under review of female- and male-authored articles. **A)** Correlation between the median time under review of articles with a female first author and articles with a male first author. **B)** Correlation between the median time under review of articles with a female corresponding author and articles with a male corresponding author. Each dot represents one journal. Blue lines correspond to female- and male-authored articles spending the same amount of time under review. 70.4% (A) and 73.1% (B) of the dots lie above the diagonal, indicating that female-authored articles often experience longer review times. This graph is based on Dataset 2, removing journals with less than 10 articles with a female first author or with less than 10 articles with a male first author **(A)**, or removing journals with less than 10 articles with a female corresponding author or with less than 10 articles with a male corresponding author **(B)**. The data underlying this Figure can be found in Zenodo (https://doi.org/10.5281/zenodo.17796183).

Next, we investigated these trends within the articles published in each journal separately. We first focused our analysis on the 5,611 journals with at least 10 articles with a female first author and 10 articles with a male first author. For each of these journals, we calculated the median time under review of articles with female first authors (*T*_F1_) and the median time under review of articles with male first authors (*T*_M1_). Across all 5,611 journals, the median values of *T*_F1_, *T*_M1_, and *T*_F1_/*T*_M1_ ratio were 104 days, 98 days, and 1.051, respectively. *T*_F1_ was higher than *T*_M1_ (indicating that female-authored articles tend to spend longer under review) in 3,951 of the journals, lower than *T*_M1_ (indicating that male-authored articles tend to spend longer under review) in 1,465 of the journals, and equal to *T*_M1_ (indicating that female- and male-authored articles spend the same amount of time under review) in 195 of the journals. These results indicate that female-authored articles experienced longer review times in 70.4% of the journals (Fig 3A). The correlation between the fraction of female authors and time under review was positive (*ρ* > 0) in 4,068 journals, significantly positive (*ρ* > 0 and *P* < 0.05) in 1,472 journals, negative (*ρ* < 0) in 1,543 journals, and significantly negative (*ρ* < 0 and *P* < 0.05) in 163 journals.

Next, we focused on the 2,901 journals with at least 10 articles with a female corresponding author and 10 articles with a male corresponding author. For each of these journals, we calculated the median time under review of articles with female corresponding authors (*T*_FC_) and the median time under review of articles with male first authors (*T*_MC_). Across all 2,901 journals, the median values of *T*_FC_, *T*_MC_, and *T*_FC_/*T*_MC_ ratio were 113.5 days, 105 days, and 1.056, respectively. *T*_FC_ was higher than *T*_MC_ (indicating that female-authored articles tend to spend longer under review) in 2,121 of the journals, lower than *T*_MC_ (indicating that male-authored articles tend to spend longer under review) in 696 of the journals, and equal to *T*_MC_ (indicating that female-authored and male-authored articles spend the same amount of time under review) in 84 of the journals. These results indicate that female-authored articles experienced longer review times in 73.1% of the journals (Fig 3B). The correlation between the fraction of female authors and time under review was positive (*ρ* > 0) in 2,201 journals, significantly positive (*ρ* > 0 and *P* < 0.05) in 987 journals, negative (*ρ* < 0) in 700 journals, and significantly negative (*ρ* < 0 and *P* < 0.05) in 95 journals.

### Differences in publication date do not explain why female-authored articles spend longer under review

Given that female representation among authors of biomedical and life science research articles significantly increased over time (Fig 1; refs. [3,4,44,48]), female-authored articles indexed in the PubMed database are expected to be, on average, more recent than male-authored articles. In addition, previous work has shown that, in top Economics journals and in some Evolutionary Biology journals, the average amount of time that articles spent under review has increased in the last decades [44,49]. If the longer time under review for recent articles was generalizable to most biomedical and life sciences journals, that could explain why female-authored articles in our dataset spent on average longer under review.

**Fig 4:**
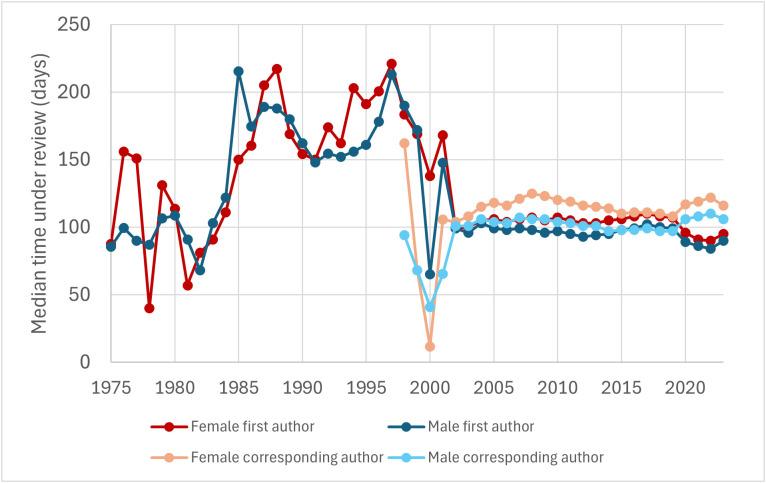
Evolution of the median time under review of articles with female first authors, male first authors, female corresponding authors, and male corresponding authors. This graph is based on Dataset 2 (excluding articles published before 1975). 97.4% of articles in Dataset 2 were published between 2003 and 2024. The strong fluctuations among articles published before 2003 may be due to much smaller sample sizes. Since corresponding authors were inferred based on availability of email addresses, we only computed the percentage of female corresponding authors since the 1995–1999 quinquennium. The data underlying this Figure can be found in Zenodo (https://doi.org/10.5281/zenodo.17796183).

To test this possibility, we classified articles according to their year of publication, and calculated *T*_F1_, *T*_M1_, *T*_FC_, and *T*_MC_ for each year separately. We did not observe a positive correlation between articles’ time under review and their date of publication (Fig 4). Indeed, *T*_F1_, *T*_M1_, *T*_FC_, and *T*_MC_ did not substantially change between 2003 and 2024 (a period that concentrates 97.4% of articles in Dataset 2). Times under review substantially fluctuated in previous years (probably due to much smaller sample sizes), but they did not increase over time. These observations indicate that the previously observed slow-down in the peer review process in some journals [44,49] does not apply to articles indexed in PubMed as a whole.

In addition, for all years between 2003 and 2024, articles with a female first author spent significantly longer under review than articles with a male first author (Mann–Whitney’s *U* test, *P* < 0.05; Fig 4), articles with a female corresponding author spent significantly longer under review than articles with a male corresponding author (Mann–Whitney’s *U* test, *P* < 0.05; Fig 4), and articles’ fraction of female authors positively correlated with articles’ time spent under review (*ρ* > 0, *P* < 0.05). These results indicate that the differences in the time spent under review by female- and male-authored articles are not due to differences in articles’ dates of publication.

### Female-authored articles are slightly longer, but that does not explain why they spend longer under review

Out of the 7,758,839 articles in Dataset 2, we could retrieve the number of pages for 4,513,913, including 1,480,160 with a female first author, 2,078,633 with a male first author, 642,839 with a female corresponding author, and 1,306,053 with a male corresponding author. Articles with a female corresponding author were slightly longer (median: 9 pages) than those with a male corresponding author (median: 8 pages; Mann–Whitney’s *U* test, *P* < 10^−290^). Also, significant differences were detected between articles with a female corresponding author (median: 8 pages) and articles with a male corresponding author (median: 8 pages; Mann–Whitney’s *U* test, *P* = 3.93 × 10^−99^). In addition, articles’ fraction of female authors weakly but positively correlates with articles’ number of pages (*ρ* = 0.050, *P* < 10^−290^), and a separate analysis of each journal confirms that female-authored articles tend to be slightly longer than male-authored published in the same journal (see S1 Text)

**Fig 5:**
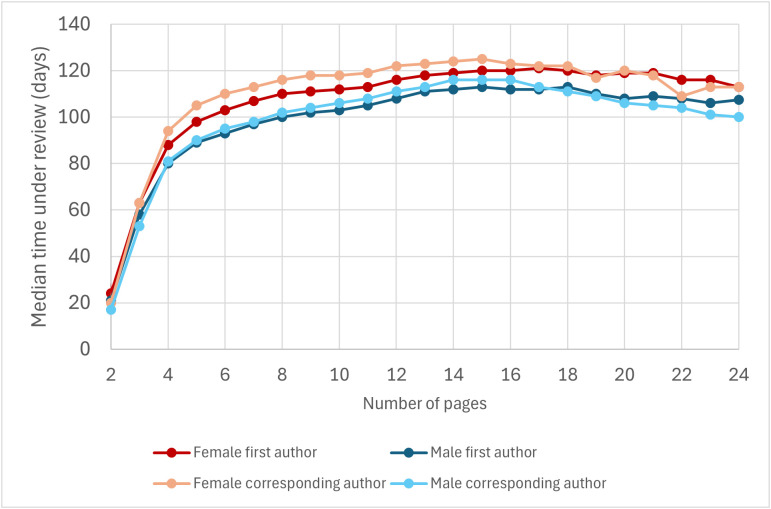
Median time under review of articles with female first authors, male first authors, female corresponding authors, and male corresponding authors as a function of their length. This graph is based on Dataset 2 (excluding 1–page articles, articles that were longer than 24 pages, and those whose length was not available). 99.1% of the articles in the dataset have 2–24 pages. The data underlying this Figure can be found in Zenodo (https://doi.org/10.5281/zenodo.17796183).

If longer articles spend longer under review, that could explain why female-authored articles tend to spend longer under review. To test this possibility, we classified articles according to their length, and calculated *T*_F1_, *T*_M1_, *T*_FC_, and *T*_MC_ for each length class (each length class was composed by all articles of a certain length, e.g., 10 pages) separately. We did not observe a positive correlation between articles’ length and how long they spent under review. Instead, we observed a non-monotonic relationship: among articles with 2–15 pages, longer articles tend to spend longer under review, whereas among longer articles, longer articles tend to experience shorter review times (Fig 5).

In addition, for all length classes between 2 and 24 pages (a range that concentrates 99.1% of the articles in Dataset 2 with available length), articles with a female first author spent significantly longer under review than articles with a male first author (Mann–Whitney’s *U* test, *P* < 0.05; Fig 5) and articles’ fraction of female authors positively correlated with articles’ time spent under review (*ρ* > 0, *P* < 0.05). In addition, for all length classes between 2 and 26 pages (99.4% of the articles in Dataset 2 with available length), articles with a female corresponding author spent significantly longer under review than articles with a male corresponding author (Mann–Whitney’s *U* test, *P* < 0.05; Fig 5). Outside these ranges, trends are less clear due to small sample sizes. These results indicate that the differences in the time under review spent by female- and male-authored articles are not due to differences in their length.

### Articles with female corresponding authors are slightly more readable, but that does not explain why they spend longer under review

Out of the 7,758,839 articles in Dataset 2, we could retrieve the abstract text for 7,609,358. For each of these abstracts, we computed five readability scores: Flesch Reading Ease Score [50], Flesch–Kincaid Readability Score [51], Gunning’s Fog Index [52], Simple Measure of Gobbledygook (SMOG) [53], and Dale–Chall Readability Formula [54]. When we analyzed all 7,609,358 articles simultaneously, we found significant differences between the scores of articles with a female corresponding author and the five scores of articles with a male corresponding author: all five scores indicated that articles with a female corresponding author tend to be slightly more readable (nonetheless, differences were very small: the biggest difference in the median scores was of only 5.7‰; Table 1). We corroborated this trend by analyzing the abstracts of articles published in each journal separately, and calculating the medians for all five scores for abstracts with a female versus a male corresponding author. A total of 2,885 journals had indexed at least 10 abstracts with a female corresponding author and 10 abstracts with a male corresponding author. Out of these journals, abstracts with female corresponding authors were more readable than abstract with male corresponding authors in 1,445 (50.1%), 1,542 (53.4%), 1,562 (54.1%), 1,483 (51.4%), and 1,704 (59.1%) according to the median Flesch, Flesch–Kincaid, Fog, SMOG, and Dale–Chall scores, respectively, indicating a subtle tendency for abstracts of articles with female corresponding authors to be more readable.

**Table 1:** Readability scores of abstracts by female and male authors.

Readability score	First author					Corresponding author					Correlation readability score-fraction of female authors	
	Female name		Male name		*P*-value^a^	Female name		Male name		*P*-value^a^		
	*n*	Median	*n*	Median		*n*	Median	*n*	Median		ρ	*P*-value^b^
Flesch	2,526,791	40.99	3,338,103	41.19	**2.73 × 10** ^** − 74**^	958,090	41.02	1,910,602	40.87	**4.79 × 10** ^** − 27**^	−0.0029	**5.37 × 10** ^** − 16**^
Flesch–Kincaid	2,526,791	13.22	3,338,103	13.21	**3.05 × 10** ^** − 10**^	958,090	13.21	1,910,602	13.28	**5.22 × 10** ^** − 85**^	−0.0037	**3.05 × 10** ^** − 24**^
Gunning’s Fog	2,526,791	16.81	3,338,103	16.80	**3.76 × 10** ^** − 3**^	958,090	16.79	1,910,602	16.88	**5.64 × 10** ^** − 152**^	−0.0058	**3.43 × 10** ^** − 58**^
SMOG	2,526,791	14.96	3,338,103	14.94	**4.28 × 10** ^** − 17**^	958,090	14.94	1,910,602	15.02	**1.20 × 10** ^** − 126**^	−0.0034	**1.03 × 10** ^** − 20**^
Dale–Chall	2,526,791	12.21	3,338,103	12.23	**7.60 × 10** ^** − 84**^	958,090	12.12	1,910,602	12.28	**<10** ^** − 290**^	−0.0284	**<10** ^** − 290**^

*P*-values lower than 0.05 are shown in bold face.

^a^*P*-values corresponding to the Mann–Whitney’s *U* test.

^b^*P*-values corresponding to Spearman’s correlation test.

However, differences between articles with a female first author and articles with a male first author were inconsistent: according to the Flesch, Flesch–Kincaid, Fog, and SMOG scores, articles with a male first author are slightly more readable; conversely, according the Dale–Chall score, articles with a female first author are slightly more readable (nonetheless, differences were again very small: the biggest difference was of only 4.9‰; Table 1). Separate analysis of abstracts published in different journals also resulted in inconsistent results: out of the 5,579 journals that published at least 10 abstracts with a female fist author and 10 abstracts with a male first author, female-authored articles were more readable in 2,650 (47.5%), 2,821 (50.6%), 2,937 (52.6%), 2,721 (48.8%), and 2,799 (50.2%) according to the median Flesch, Flesch–Kincaid, Fog, SMOG, and Dale–Chall scores, respectively, indicating that different scores produce different results.

**Fig 6:**
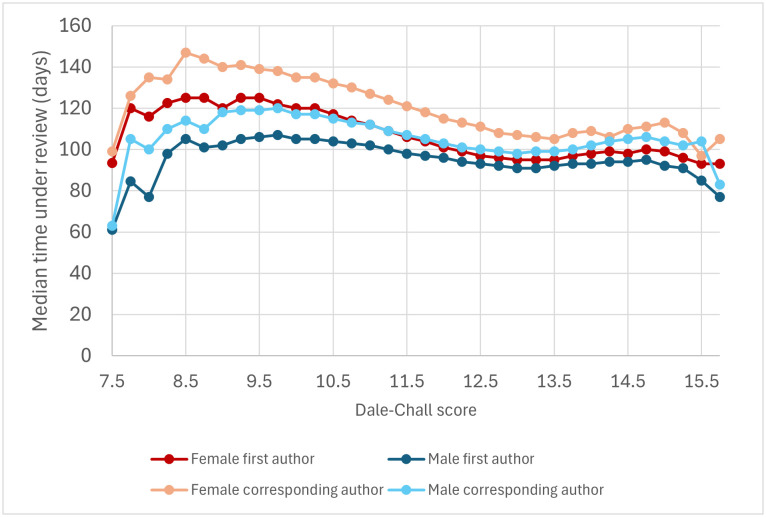
Median time under review of articles with female first authors, male first authors, female corresponding authors, and male corresponding authors as a function of their abstract’s Dale–Chall readability score. This graph is based on Dataset 2, excluding articles with a missing abstract and those with abstracts’ readability scores outside the range [7.5, 16). Each category included abstracts with similar scores, down to a quarter of a unit. Each group is labeled according to its lowest value—e.g., abstracts with a score in the range [10, 10.25) were included in the “10” category. 99.96% of the articles in the dataset have abstracts with a score in the range [7.5–16). Highly readable texts tend to exhibit low Dale–Chall scores. The data underlying this Figure can be found in Zenodo (https://doi.org/10.5281/zenodo.17796183).

An analysis of abstracts published in top Economics journals has suggested that one of the reasons why female-authored articles tend to spend longer under review is that female researchers tend to devote more effort to making their articles more readable [38]. Is that the case for articles indexed in PubMed? To test this possibility, we binned abstracts according to their Dale–Chall score. Each category included abstracts with similar scores, down to a quarter of a unit (10.000–10.249, 10.250–10.499, etc.). For all categories between 7.5 and 16 (categories that concentrate 99.96% of the abstracts in Dataset 2), articles with a female first author spent significantly longer under review than articles with a male first author (Mann–Whitney’s *U* test, *P* < 0.05; Fig 6). For all categories between 8.25 and 15.25 (99.8% of the abstracts in Dataset 2), articles with a female corresponding author spent significantly longer under review than articles with a male corresponding author (Mann–Whitney’s *U* test, *P* < 0.05; Fig 6). For all categories between 7.5 and 16.5 (99.97% of the abstracts in Dataset 2), articles’ fraction of female authors exhibited a significantly positive correlation with the amount of time that they spent under review (*ρ* > 0, *P* < 0.05). Similar results were obtained when abstracts were binned according to their Flesch, Flesch–Kincaid, Fog, and SMOG scores (S2–S5 Figs). These results indicate that female-authored articles tend to spend longer under review, regardless of their readabilities.

### Female-authored articles have slightly more co-authors, but that does not explain why they spend longer under review

Previous analyses of Evolutionary Biology articles suggested that female-authored articles tend to have more co-authors than male-authored articles, and that articles with more co-authors tend to spend longer under review [44]. If these trends are generalizable to most biomedical and life sciences journals, they could explain why female-authored articles tend to spend longer under review. We found significant differences between the number of authors of articles with a female first author and those with a male first author (Mann–Whitney’s *U* test, *P* < 10^−290^), and between the number of authors of articles with a female corresponding author and those with a male corresponding author (*P* = 1.37 × 10^−147^); however, differences were small and all four categories exhibited a median of 5 authors. The fraction of female authors weakly but positively correlated with the number of authors (*ρ* = 0.072, *P* < 10^−290^), and a separate analysis of each journal confirms that female-authored articles tend to have slightly more co-authors than male-authored articles published in the same journal (S1 Text).

**Fig 7:**
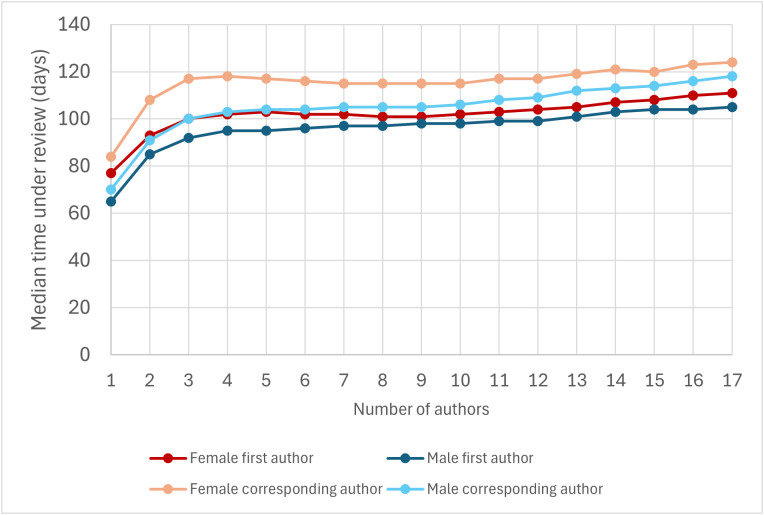
Median time under review of articles with female first authors, male first authors, female corresponding authors, and male corresponding authors as a function of their number of authors. This graph is based on Dataset 2 (excluding articles with more than 17 authors). 97.9% of the articles in the dataset have 1–17 authors. The data underlying this Figure can be found in Zenodo (https://doi.org/10.5281/zenodo.17796183).

If articles with more authors tend to spend longer under review, that could explain why female-authored articles tend to spend longer under review. To test this possibility, we classified articles according to their number of authors, and calculated *T*_F1_, *T*_M1_, *T*_FC_, and *T*_MC_ for each number of authors separately. We did observe a clear positive relationship between articles’ number of authors and how long they spend under review, especially among articles with 1–4 authors; for articles with more than 4 authors, the relationship was much less clear (Fig 7). To remove the potential effect of the number of authors, we classified articles according to their number of authors and analyzed each category separately. For all categories between 1 and 20 authors (a range that concentrates 98.8% of the articles in Dataset 2), articles with a female first author spent significantly longer under review than articles with a male first author (Mann–Whitney’s *U* test, *P* < 0.05; Fig 7). In addition, for all categories between 1 and 17 articles (97.9% of the articles in Dataset 2), articles with a female corresponding author spent significantly longer under review than articles with a male corresponding author (Mann–Whitney’s *U* test, *P* < 0.05; Fig 7), and articles’ fraction of female authors positively correlated with articles’ time spent under review (*ρ* > 0, *P* < 0.05). These results indicate that the differences in the amount of time spent under review by female- and male-authored articles are not due to differences in their number of authors.

### Female-authored articles spend longer under review in most fields, irrespective of female representation

The difference between the time spent under review by female- and male-authored articles substantially differs among journals: among the 5,611 journals with at least 10 articles with a female first author and 10 articles with a male first author, *T*_F1_/*T*_M1_ ratios ranged from 0.06 to 7.78. Similarly, among the 2,901 journals with at least 10 articles with a female corresponding author and 10 articles with a male corresponding author, *T*_FC_/*T*_MC_ ranged from 0.16 to 4.60. We considered the possibility that the observed trend that female-authored articles spend longer under review could be more pronounced for fields in which women are more underrepresented. However, among the 5,611 journals with at least 10 articles with a female first author and 10 articles with a male first author, journals’ *T*_F1_/*T*_M1_ ratios did not correlate with journals’ fraction of female authors (*ρ* = 0.021, *P* = 0.110). Similarly, among the 2,901 journals with at least 10 articles with a female corresponding author and 10 articles with a male corresponding author, *T*_FC_/*T*_MC_ ratios did not correlate with journals’ fraction of female authors (*ρ* = −0.012, *P* = 0.532).

We next used the National Library of Medicine’s Broad Subject Terms (*n* = 124) to group journals into different categories, and we calculated *T*_F1_, *T*_M1_, *T*_FC_, and T_MC_ for articles within each category. Among the 120 categories with at least 100 articles with a female first author and 100 articles with a male first author, *T*_F1_/*T*_M1_ ratios ranged from 0.89 (for “Biophysics”) to 1.27 (for “Biotechnology”). T_F1_/T_M1_ ratios were lower than 1 for 13 categories (differences were significant for 10 of these categories according to the Mann–Whitney’s *U* test, indicating that male-authored articles in these categories spend significantly longer under review), equal to 1 for 2 categories (indicating that female- and male-authored articles spend the same amount of time under review), and higher than 1 for 104 categories (differences were significant for 95 of these categories, indicating that female-authored articles in these categories tend to spend longer under review; S1 Table). Similarly, among the 118 categories with at least 100 articles with a female corresponding author and 100 articles with a male corresponding author, *T*_FC_/*T*_MC_ ratios ranged from 0.91 (for “Clinical Laboratory Techniques”) to 1.38 (for “Audiology”). *T*_FC_/*T*_MC_ ratios were lower than 1 for 9 categories (differences were statistically significant for 6 of these categories), equal to 1 for 2 categories, and higher than 1 for 106 categories (differences were statistically significant for 93 of these categories). Neither *T*_F1_/*T*_M1_ ratios (*ρ* = 0.094, *P* = 0.302) nor *T*_FC_/*T*_MC_ ratios (*ρ* = 0.048, *P* = 0.600) significantly correlate with categories’ fraction of female authors. Taken together, these results indicate that, in most fields, female-authored articles tend to spend longer under review, and that the gender gap does not depend on how well women are represented in each field.

### Female-authored articles spend longer under review in most countries/territories, irrespective of female representation and GDP per capita

**Fig 8:**
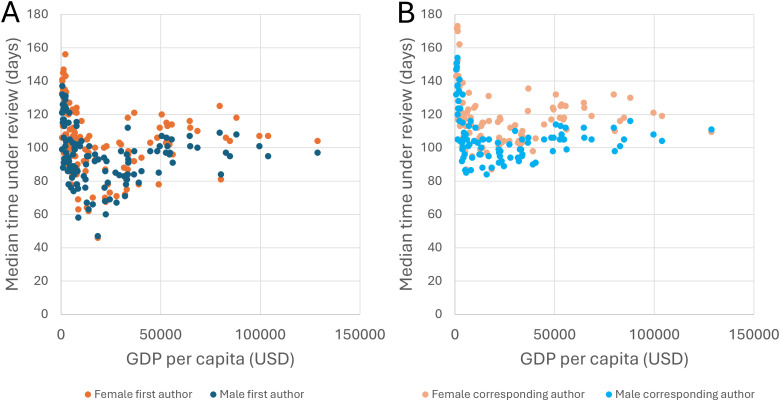
Correlation between countries’ gross domestic products (GDP) per capita and the median time under review of articles by first (A) and corresponding (B) authors based in the country. Each country is represented by two dots in each of the graphs. The data underlying this Figure can be found in Zenodo (https://doi.org/10.5281/zenodo.17796183).

Out of the 7,758,839 articles in Dataset 2, we could extract the country/territory of the first author for 7,597,974, and the country/territory of the corresponding author for 3,680,528. We first classified articles according to the country/territory to which the first author is affiliated, and calculated a separate *T*_F1_, *T*_M1_, and *T*_F1_/*T*_M1_ ratio for each country/territory. Among the 126 countries/territories with at least 100 articles with a female first author and 100 articles with a male first author, the *T*_F1_/*T*_M1_ ratio ranged from 0.78 (for Brunei) to 1.50 (for Paraguay). The *T*_F1_/*T*_M1_ ratio was lower than 1 for 12 countries/territories (however, differences were not significant for any of these 12 countries/territories according to the Mann–Whitney’s *U* test), equal to 1 for 4 countries/territories (indicating than female and male authors based in these countries/territories tend to experience identical review times), and higher than 1 for 110 countries/territories (differences were significant for 74 of these countries/territories, indicating that female authors based in these countries/territories tend to experience significantly longer review times; S2 Table). Countries’ gross domestic products (GDP) per capita negatively correlated with both *T*_F1_ (*ρ* = −0.389, *P* = 8.63 × 10^−6^) and *T*_M1_ (*ρ* = −0.331, *P* = 1.85 × 10^−^^4^; Fig 8A), indicating that articles with first authors based in lower-income countries tend to experience longer review times. Of note, articles with first authors based in countries classified as low-income by the World Bank spent a median time under review of 122 days (i.e., 25.8% longer than the median from the rest of the countries, i.e., 97 days). However, countries’ GDP per capita did not correlate with their *T*_F1_/*T*_M1_ ratios (*ρ* = −0.117, *P* = 0.196), indicating that the bias affects female and male authors equally. In addition, countries’ *T*_F1_/*T*_M1_ ratios did not correlate with their fraction of female authors (*ρ* = 0.010, *P* = 0.908).

We then classified articles according to the country/territory in which the corresponding author is based, and calculated a separate *T*_FC_, *T*_MC_, and *T*_FC_/*T*_MC_ ratio for each country/territory. Among the 97 countries/territories with at least 100 articles with a female corresponding author and 100 articles with a male corresponding author, the *T*_FC_/*T*_MC_ ratio ranged from 0.97 (for Malawi) to 1.38 (for Costa Rica). The *T*_FC_/*T*_MC_ ratio was lower than 1 for 4 countries/territories (differences were not significant for any of these countries/territories) and higher than 1 for 93 countries/territories (with significant differences for 77 countries/territories; S3 Table). Countries’ GDP per capita negatively correlated with both *T*_FC_ (*ρ* = −0.263, *P* = 0.010) and *T*_MC_ (*ρ* = −0.243, *P* = 0.018; Fig 8B), indicating that articles with corresponding authors based in lower-income countries tend to experience longer review times. Indeed, articles with corresponding authors based in low-income countries spent a median time under review of 140 days (i.e., 44.3% longer than the median from the rest of the countries). However, countries’ *T*_FC_/*T*_MC_ ratios did not correlate with their GDP per capita (*ρ* = 0.016, *P* = 0.876) or with their fraction of female authors (*ρ* = −0.049, *P* = 0.635).

### Female-authored articles spend longer under review after controlling for multiple factors simultaneously

Results presented in Figs 4–8 and S2–S5 suggest that female-authored articles spend longer under review regardless of their date of publication, length, readability, number of authors, or GDP per capita of the countries in which the authors are based. However, these results alone do not allow us to discard the possibility that, combined, differences in these factors might explain the differences in the times under review experienced by female- and male-authored articles. To control for all these factors simultaneously, we conducted an ANCOVA using time under review as the dependent variable and gender of the first author, gender of the corresponding author, fraction of female authors, year of publication, length, number of authors, Dale–Chall readability score, GDP per capita of the country of the first author, and GDP per capita of the country of the corresponding author as independent variables. In addition, we included the journal as a random effect. To improve the linearity of the relationships between time under review and the independent variables, we removed articles published before 2003, those with fewer than 6 pages, those with fewer than 3 authors, and those with a Dale–Chall score below 8.5. After filtering, our dataset contained 1,207,600 articles for which all variables were available and for which the genders of the first and the corresponding author could be inferred. Out of these articles, 600,000 were randomly selected and included in the analysis. None of the variables exhibited a variance inflation factor above 9. The analysis shows that having a female first author, a female corresponding author, or a large fraction of female authors significantly increases articles’ time under review. In addition, articles with many authors, long articles, and articles whose corresponding author is based in a lower-income country also tend to experience longer review times (Table 2). Surprisingly, recent articles, and those with lowly readable abstracts tend to experience shorter review times (Table 2)

**Table 2:** Factors affecting articles’ time under review (ANCOVA).

Variable	Estimate (*β*)	Std. Error	*t*-value	*P*-value
Intercept	432.80	58.06	7.45	9.00 × 10^ − 14^***
Gender first author (male)	−2.75	0.31	−8.92	4.52 × 10^ − 19^***
Gender corresponding author (male)	−3.73	0.32	−11.78	5.19 × 10^ − 32^***
Fraction of female authors	1.24	0.56	2.21	0.027*
Year of publication	−0.14	0.03	−4.80	1.61 × 10^ − 6^***
Number of authors	0.16	0.02	7.37	1.65 × 10^ − 13^***
Number of pages	0.77	0.01	7.01	2.37 × 10^ − 12^***
GDP per capita of first author’s country	−1.78 × 10^ − 5^	1.43 × 10^ − 5^	−1.24	0.214
GDP per capita of corresponding author’s country	−1.10 × 10^ − 4^	1.44 × 10^ − 5^	−7.66	1.87 × 10^ − 14^***
Dale–Chall readability score	−0.86	0.12	−6.90	5.19 × 10^ − 12^***

* *P* < 0.05; ** *P* < 0.01; *** *P* < 0.001.

Abbreviation: GDP, gross domestic products.

## Discussion

### Women are underrepresented in biomedicine and the life sciences, but their representation greatly increased in the last decades

We found that women are underrepresented among authors of articles indexed in PubMed, but that their representation greatly increased over time. Indeed, female representation steadily increased from only 2.3% in 1900–1904 to 38.7% of the authors of articles published in 2020–2024 (Fig 1). Both the current level of female representation and the increase over time that we observed are consistent with the results of previous analyses of the PubMed database [4], as well as with analyses of articles of specific fields [3,4,6,44,48].

Also consistent with previous results [4,15,44,47,55], we found that female authors are better represented among first authors than among all authors, and less well represented among corresponding authors than among all authors (Fig 1). For instance, women account for 43.5% of the first authors and 33.4% of the corresponding authors of articles published in 2020–2024 (Fig 1). These observations may have at least three explanations. First, since female representation in biomedicine and the life sciences has increased over time and female researchers are more likely to leave academia than male researchers [7,8], we expect that female authors in our dataset will be, on average, less senior than male authors in our dataset [56]. In biomedicine and the life sciences, corresponding authors tend to be the researchers that supervised the research (these are often senior researchers, especially group leaders; refs. [46,47]). Second, women are less likely than men to be well funded [7,15], which limits their ability to lead research projects [27]. Third, female researchers may be less likely to request or to be offered corresponding authorship [4,34,57].

It should be noted that female representation among authors of a set of research articles depends not only on the percentage of active researchers in the field that are female, but also on the relative publication rates of female researchers, which tends to be lower than those of male researchers [26–28]. Therefore, the percentage of female researchers in biomedicine and the life sciences is expected to be higher than the numbers presented in Fig 1.

### Female-authored articles tend to spend longer under review

To our knowledge, we have conducted the largest analysis to date of the amount of time that research articles spend under review—previous analyses have focused on hundreds or thousands of articles [37,39–44,49], whereas our analysis encompasses millions of articles. We provide unequivocal evidence that female-authored articles tend to spend longer under review than male-authored articles. The median time under review was 7 days (7.4%) longer for articles with a female first author than for articles with a male first author, 13 days (12.7%) longer for articles with a female corresponding author than for articles with a male corresponding author, 15 days (14.6%) longer for articles with a female first author and a female corresponding author than for articles with a male first author and a male corresponding author, and 9 days (10.0%) longer for articles authored by all-female teams than for articles authored by all-male teams (Fig 2). In addition, articles’ fraction of female authors positively correlates with the amount of time that they spend under review. The gender gap holds after controlling for a number of confounding factors, including the specific journal (Fig 3) and articles’ date of publication (Fig 4), number of pages (Fig 5), and number of co-authors (Fig 7), indicating that it is not mediated by these factors.

Even though, for each manuscript, time under review is only 7–15 days longer for female- than for male-authored articles (Fig 2), accumulated over a woman’s career, these delays can be very substantial. For instance, for every 50 papers published by a female author, she will have spent on average 350–750 days longer than her male counterparts waiting for reviews and editorial decisions and/or revising manuscripts. These numbers should be taken with caution since they don’t account for the time that manuscripts may spend under consideration by other journals before they land their final publication outlet, and they assume that authors never have two or more manuscripts under review at the same time, which is not realistic. Nevertheless, the longer review times experienced by female researchers may explain, at least to some extent, why they publish, on average, at lower rates than their male counterparts [26–28].

A combination of factors may explain why female-authored articles tend to spend longer under review. First, editors and referees may be prejudiced against female-authored articles, being more likely to be critical of their content. This may be due to them holding inadequate stereotypes against female researchers, to them being prejudiced against the institutions to which female authors are affiliated (women are underrepresented at top research institutions; refs. [58,59]), or to them being relatively unfamiliar with the topics covered in female-authored articles (female researchers tend to be better represented in certain subfields). Second, female researchers may take longer to revise and resubmit their manuscripts due to extrinsic (e.g., more duties at home and/or higher teaching and/or service loads; refs. [12,13]) or intrinsic factors (e.g., more perfectionism, self-doubt, or risk-aversion; refs. [60–65]). Third, as argued above, female authors in our dataset are expected to be, on average, less experienced than male authors in our dataset. While our comparisons of articles with female versus male corresponding authors provides some level of control for seniority—corresponding authors tend to be experienced researchers—, it is still possible that female corresponding authors in our dataset are on average somewhat less experienced than male corresponding authors in our dataset. Due to the complexity of the issue, it is hard to measure the relative impact of the different factors or to propose a single solution. Double-blind peer review (i.e., a peer review system in which the referees do not know the identity or the affiliation of the authors) is expected to partially eliminate the first factor, but it is not expected to alleviate the other two. To specifically test whether referees tend to spend longer evaluating female-authored manuscripts because they hold negative stereotypes against female researchers (rather than because they are prejudiced against their institutions or because they are unfamiliar with the topics covered by their research), an experiment could be conducted in which the same manuscript would be evaluated by two groups of referees (for one group the manuscript would be signed by female authors and for the other groups it would be signed by male authors).

Previous studies of articles published in top Economics journals suggest that editors and referees are biased against female-authored manuscripts. Card and colleagues [43] found that, among articles published in four journals, female-authored ones tend to be more cited (a proxy for higher quality) than male-authored ones, suggesting that editors and/or referees hold female-authored articles to higher standards. In addition, by comparing the abstracts of 9,117 articles published in four journals with their preprint versions, Hengel [38] showed that female economists tend to rewrite their manuscripts more substantially than male economists during the peer review process to make them more readable, suggesting that editors and/or referees are more likely to ask authors to significantly rewrite their manuscripts when they are female. Moreover, by analyzing administrative data from 33 Economics and Finance journals, Alexander and colleagues [39] showed that female-authored manuscripts spend longer in all the stages of the peer review process (i.e., they spend longer being evaluated by the referees and being revised by the authors) and require more rounds of peer review, also suggesting a more rigorous review process. However, it is unclear to what extent these trends apply to articles published in fields other than Economics. In addition, even if editors and/or reviewers of biomedical and life sciences journals were often biased against female-authored work, a contribution of other factors cannot be discarded.

Our analyses show that female-authored articles spend longer under review in most, but not all, biomedical and life sciences fields. Actually, the trend is reversed in a minority of fields: in the fields of “Biophysics,” “Hospitals,” “Biology,” “Molecular Biology,” “Chemistry,” “Women’s Health,” “Genetics,” “Health Services,” “Environmental Health,” and “Computational Biology,” articles with a female first author experience significantly *shorter* review times than articles with a male first author (Mann–Whitney’s *U* test, *P* < 0.05; S1 Table). Similarly, in the fields of “Clinical Laboratory Techniques,” “Hospitals,” “Women’s Health,” “Health Services,” and “Biology,” articles with a female corresponding author experience significantly *shorter* review times than articles with a male corresponding author (Mann–Whitney’s *U* test, *P* < 0.05; S1 Table). This may explain why Alvarez-Ponce and Vesper [44] did not find substantial differences between the amount of time spent under review by female- and male-authored Evolutionary Biology research articles—indeed, Evolutionary Biology could be classified within the “Biology” category, and it strongly intersects with “Molecular Biology,” “Genetics,” and “Computational Biology.” Conversely, Hagan and colleagues [37] found that, among manuscripts submitted to the journals of the American Society for Microbiology, female-authored ones took on average 1–9 days longer to be accepted than male-authored ones. This is consistent with the fact that the many categories for which we found that female-authored articles spend longer under review include “Microbiology,” “Bacteriology,” and “Virology” (S1 Table).

The trend also affects authors from most countries. Even though the differences in time under review of female- versus male-authored articles are not statistically significant for all countries, the countries for which differences are significant are much better represented in our dataset than the countries for which differences are not significant (median number of articles: 22,685.5 versus 1,388 for S2 Table, and 12,304 versus 1,107 for S3 Table), suggesting that differences are not significant for some countries due to limited statistical power. Remarkably, authors from lower-income countries tend to experience longer review times. This could be due, at least in part, to editors and/or referees holding their manuscripts to higher standards, to them having fewer resources to address the requested modifications (which often require additional experiments or data generation), or to language barriers [66]. Nonetheless, female and male authors from lower-income countries seem to be affected equally by these additional delays.

### Readability scores

By applying five readability formulas to the abstracts of 9,117 articles published in four Economics journals, Hengel [38] found that, on average, female-authored abstracts are more readable than male-authored ones, and that female economists improve the readability of their abstracts as they become more experienced, whereas male economists do not. In addition, by comparing these published abstracts with their preprint versions, she found that female economists rewrite their abstracts more substantially than male economists during the peer review process to make them more readable, which may explain why female-authored articles spend longer under review (of note, the readability of abstracts positively correlates with the readability of other sections of articles, and writing highly readable texts takes time; refs. [38,67–70]).

We applied the same five readability formulas to 7,609,358 abstracts indexed in PubMed. We found that abstracts of articles with female corresponding authors were slightly more readable than those of articles with male corresponding authors. However, the differences between abstracts with female versus male first authors were inconsistent, with some readability scores indicating that female-authored articles were slightly more readable and with other scores indicating the opposite (Table 1). Our observations are consistent with the scenario proposed by Hengel [38], in which female researchers become better writers over time whereas male researchers do not. This may explain why differences between abstracts authored by female versus male first authors are unclear (junior researchers tend to be first authors) whereas differences between abstracts authored by female versus male corresponding authors are more clear (corresponding authors tend to be experienced authors). However, the differences that we found are much smaller (0.48–5.7‰; Table 1) than those found by Hengel (1%–6%). In addition, female-authored articles spend significantly longer under review than male-authored articles regardless of their readability (Figs 6 and S2–S5), indicating that, in biomedicine and the life sciences, the gender gap in submission-to-acceptance times is not due to female researchers investing more time in improving the readability of their manuscripts.

### Limitations of our study

Our study is subject to some limitations. First, our dataset is subject to some missing or incomplete data: for some of the articles, some information is unavailable or only partially available in the PubMed database. For instance, submission and acceptance dates are missing for some articles, and the gender could not be inferred for a substantial fraction of authors because they had unisex names or names that were not present in the Genderize database, or because only their first names’ initials were provided in the PubMed database. In addition, we determined articles’ corresponding authors based on the availability of email addresses in the PubMed database, which prevented us from determining the corresponding authors of articles published before the internet era. Nonetheless, even after filtering out articles with missing data (different filters were applied depending on the specific analysis), the number of articles available for analysis was still very large.

Second, first names are not perfect predictors of gender; therefore, some women may have misclassified as men and some men may have been misclassified as women. However, the method that we used to infer gender has been validated by comparing its results against reference lists of scientists of known gender [4,44,71,72]. In addition, our estimates of the fraction of researchers that are female are similar to those produced by previous studies, including studies in which gender was self-reported by researchers or determined through internet searches [4,34,44,48,73,74].

Third, some of the previous studies evaluating the differences in the time spent under review by female- and male-authored articles have included variables that are hard to obtain and/or that require manual curation, including the authors’ seniority, the gender of the editors, whether the journal uses double-blind peer review, or whether women gave birth, were on maternal leave or had young children during the peer review period [38,41]. Other studies have included variables that are not publicly available, such as the time spent by manuscripts in the different steps of the peer review process or the degree of expertise of the referees [39]. However, given the large number of articles and journals analyzed in our study, including such variables would have been unfeasible.

### Conclusions and future directions

By analyzing millions of research articles indexed in the PubMed database, we show that female-authored articles tend to spend longer under review than male-authored articles, and that the trend is robust to controlling for several potentially confounding factors. Even though the gender gap is pervasive across biomedicine and the life sciences, it does not affect all fields equally, being absent and even reversed in a minority of disciplines. This raises questions about whether a gender gap in acceptance times is present in other, largely unexplored disciplines such as Mathematics and Engineering. It also raises questions about what factors contribute to or alleviate the gender gap—what can we learn from fields in which female authors do not experience longer review times? Continuing to document the different dimensions of the gender gap in academia and investigating its reasons may help the academic community devise mitigating strategies.

## Methods

### Data compilation

We downloaded PubMed’s 2024 “annual baseline” dataset from https://ftp.ncbi.nlm.nih.gov/pubmed/baseline/. This dataset contains the information of all articles indexed until the end of 2023 (36,555,430 articles published in 36,388 biomedical and life sciences academic journals). For each article, we extracted the following information (if available): PubMed ID, journal name, article title, article page range, first (given) names of all authors, affiliations of all authors, publication type (Journal Article, Letter, etc.), submission date, acceptance date, publication date, and abstract text.

From this information, we derived the following information for each article: (1) Gender of each author: each author was classified as “probably female,” “probably male,” “with unisex name,” or “of unknown name” based on their first name as described below. (2) Fraction of female authors: the number of authors classified as “probably female” divided by the number of authors classified as “probably female” plus the number of authors classified as “probable male”. (3) Country/region of each author: this information was extracted from each author’s affiliation as described below. (4) List of corresponding authors: these were those whose affiliations included an email address. (5) Amount of time that the article spent under review: the number of days intervened from submission to acceptance. (6) Number of pages: this was only inferred when the page range consisted of two numbers separated by a dash, e.g., “100–110”. Page ranges including letters were excluded. Page ranges consisting of only one number (e.g. “100”) were also excluded because, for many journals, a single number indicates the article number rather than a page number. (7) Number of authors. (8) Five readability scores, as described below.

### Gender inference

We acknowledge that not all authors identify as male or female, and that other gender identities exist. We also acknowledge that names are not perfect predictors of people’s gender. However, for the purpose of this study, we classified authors as “probably female,” “probably male,” “with unisex name,” or “of unknown gender” based on their first (given) names. Throughout our article, we use the terms “female,” “male,” “woman,” and “man” to denote gender. From each name, special characters (other than hyphens) were removed or converted to the most similar English alphabet letter. Names were then searched in the Genderize.io server (https://genderize.io/, last accessed on August 18, 2024). This server provides information for >200,000 unique names, and has been shown to perform very well in inferring scientists’ names [4,44,71,72].

Authors were deemed as “probably female” if ≥90% of the people recorded in the Genderize database with that name were female, or as “probably male” if ≥90% of the people recorded in the database with that name were male. Authors were deemed as “with unisex name” if the name was present in the database but neither gender represented ≥90% of the people recorded in the database with that name, or as “of unknown gender” if their name was not present in the Genderize database or if only their initials were available in the PubMed database.

Recent work in our lab verified that these criteria produce accurate predictions using internet searches: a random sampling of authors of Evolutionary Biology articles showed that authors inferred to be female were indeed female 98.4% of the time, and that authors inferred to be male were indeed male 99.2% of the time [44]. Even though our gender predictions are not perfect, they are probably good predictors of authors’ “perceived genders”—i.e., the gender that editors and referees tend to attribute to them based on their name.

Each article was classified in two non-exclusive ways. First, articles were classified according to the inferred gender of the first author. Second, articles were classified according to the inferred gender of the corresponding author. If an article had more than one corresponding author, the last one was retained. We focused on the these authorship positions because, in most biomedical and life science fields, they tend to be occupied by the person that conducted most of the work (first author) and by the main supervisor (last corresponding author), and are thus the positions that tend to confer the most prestige [46,47].

### Country/territory inference

Authors’ affiliations were preprocessed by removing email addresses and any appearance of the “(“, “)”, “.”, “,”, “;” and “-” characters. For each author, we extracted their country/territory of affiliation by searching their affiliation for the presence of country/region names. We obtained a list of country/territory names from the PyCountry 24.6.1 Python library, and augmented it by adding countries’ alternative names, including country names in their respective local languages (e.g., “Deutschland” or “Belgique”) and country’s three-letter codes. Since addresses in the United States and Canada often include the state’s name or two-letter code, but not the country’s name or three-letter code, we added all state names and two-letter codes (e.g., “Nevada” and “NV”) to our list. To be considered a match, country/state names/codes needed to match full words or sets of consecutive words in the affiliation. If a given affiliation matched multiple country/state names, the name whose match was closest to the end of the affiliation was retained, in case the street name may contain a country or a state name. If the affiliation contained the word “Georgia”, further steps were taken to determine whether the author was affiliated to an institution at the State of Georgia (United States) or at the Republic of Georgia. First, we determined whether the affiliation contained the name of any of the municipalities or research institutions in the State of Georgia, in which case the author was assigned to the United States. If not, or if the affiliation contained the words “Agmashenebeli,” “Kutaisi,” “Tbilisi,” “Batumi,” “Gori,” “Elavi,” “Poti,” “Zugdidi,” or “Rustavi,” the author was assigned to the Republic of Georgia.

### Abstract readability scores

Readability scores are formulas that, given a text, predict how easy it is to understand. For each abstract, we computed five readability scores using the Textatistic Python module [38]:

(1) Flesch Reading Ease Score [50]:

$$\text{2}\text{0}\text{6}.\text{8}\text{3}\text{5}-\text{1}.\text{0}\text{1}\text{5}\times \frac{words}{sentences}-\text{8}\text{4}.\text{6}\times \frac{syllables}{words}.$$

(2) Flesch–Kincaid Readability Score [51]:

$$\text{0}.\text{3}\text{9}\times \frac{words}{sentences}+\text{1}\text{1}.\text{8}\times \frac{syllables}{words}-\text{1}\text{5}.\text{5}\text{9}.$$

(3) Gunning’s Fog Index [52]:

$$\text{0}.\text{4}\times \left(\frac{words}{sentences}+\text{1}\text{0}\text{0}\times \frac{polysyllabic\;words}{words}\right).$$

(4) Simple Measure of Gobbledygook (SMOG) [53]:

$$\text{1}.\text{0}\text{4}\text{3}\times \sqrt{polysyllabic\;words\times \frac{\text{3}\text{0}}{sentences}}+\text{3}.\text{1}\text{2}\text{9}\text{1}.$$

(5) Original Dale–Chall Readability Formula [54]:

$$\text{0}.\text{1}\text{5}\text{7}\text{9}\times \text{1}\text{0}\text{0}\times \frac{difficult\;words}{words}+\text{0}.\text{0}\text{4}\text{9}\text{6}\times \frac{words}{sentences}+A.$$

Polysyllabic words are those with three or more syllables. Difficult words are those not included in Chall and Dale’s list of 3,000 familiar words understood by 80% of fourth-grade students [75]. *A* equals 0.36365 if difficult words represent more than 5% of words, and 0 otherwise. Highly readable texts tend to exhibit high Flesch Reading Ease scores. Conversely, lowly readable texts tend to score high for the other four scores, which estimate the average minimum years of schooling required to understand a text (for instance, a Dale–Chall score of 10 indicates that, on average, 10 years of schooling are required to easily understand the text).

### Additional information

To classify journals into topical categories, we obtained the list of National Library of Medicine’s Broad Subject Terms and the journals associated to each of them from https://journal-reports.nlm.nih.gov/broad-subjects/ (last accessed in March 2025). For each country, we obtained the last available estimate (in most cases, that corresponding to 2023) of its GDP per capita from the World Bank (https://data.worldbank.org/indicator/NY.GDP.PCAP.CD; last accessed on April 26, 2025).

### Statistical analyses

Mann–Whitney’s *U* tests comparing the time under review (as well as other quantities) of female- versus male-authored articles and Spearman’s correlation tests evaluating the correlation between the fraction of female authors and time under review (as well as other quantities) on different subsets of our dataset were conducted using the SciPy 1.15 Python library. Additional analyses were conducted in R 4.4.2. ANCOVA analyses were conducted using the glmer() and Anova() functions from the lme4 and car libraries, respectively. The variance inflation factor of all variables was estimated using the vif() function from the car library.

## Supporting information

S1 Fig Distribution of the percentage of female authors in 11,494 journals with at least 50 authors whose gender could be inferred. This figure is based on Dataset 1b. The data underlying this Figure can be found in Zenodo (https://doi.org/10.5281/zenodo.17796183).
(TIF)

S2 Fig Median time under review of articles with female first authors, male first authors, female corresponding authors, and male corresponding authors as a function of their abstract’s Flesch readability score. This graph is based on Dataset 2, excluding articles with a missing abstract and those with abstracts’ readability scores outside the range [7, 74). Each category included abstracts with similar scores, down to a unit. Each group is labeled according to its lowest value—e.g., abstracts with a score in the range [10, 11) were included in the “10” category. 99.48% of the articles in the dataset have abstracts with a score in the range [7, 74). The data underlying this Figure can be found in Zenodo (https://doi.org/10.5281/zenodo.17796183).
(TIF)

S3 Fig Median time under review of articles with female first authors, male first authors, female corresponding authors, and male corresponding authors as a function of their abstract’s Flesch–Kincaid readability score. This graph is based on Dataset 2, excluding articles with a missing abstract and those with abstracts’ readability scores outside the range [7, 20.25). Each category included abstracts with similar scores, down to a quarter of a unit. Each group is labeled according to its lowest value—e.g., abstracts with a score in the range [10, 10.25) were included in the “10” category. 98.65% of the articles in the dataset have abstracts with a score in the range [7, 20.25). The data underlying this Figure can be found in Zenodo (https://doi.org/10.5281/zenodo.17796183).
(TIF)

S4 Fig Median time under review of articles with female first authors, male first authors, female corresponding authors, and male corresponding authors as a function of their abstract’s Gunning’s Fog readability score. This graph is based on Dataset 2, excluding articles with a missing abstract and those with abstracts’ readability scores outside the range [9, 23.75). Each category included abstracts with similar scores, down to a quarter of a unit. Each group is labeled according to its lowest value—e.g., abstracts with a score in the range [10, 10.25) were included in the “10” category. 98.39% of the articles in the dataset have abstracts with a score in the range [9, 23.75)). The data underlying this Figure can be found in Zenodo (https://doi.org/10.5281/zenodo.17796183).
(TIF)

S5 Fig Median time under review of articles with female first authors, male first authors, female corresponding authors, and male corresponding authors as a function of their abstract’s SMOG readability score. This graph is based on Dataset 2, excluding articles with a missing abstract and those with abstracts’ readability scores outside the range [8.75, 20). Each category included abstracts with similar scores, down to a quarter of a unit. Each group is labeled according to its lowest value—e.g., abstracts with a score in the range [10, 10.25) were included in the “10” category. 98.81% of the articles in the dataset have abstracts with a score in the range [8.75, 20). The data underlying this Figure can be found in Zenodo (https://doi.org/10.5281/zenodo.17796183).
(TIF)

S1 Text Supplementary results. (PDF)

S1 Table Time under review (in days) of articles by female and male authors grouped by broad subject term. (XLSX)

S2 Table Time under review (in days) of articles by female and male authors grouped by the country/territory of the first author. (XLSX)

S3 Table Time under review (in days) of articles by female and male authors grouped by the country/territory of the corresponding author. (XLSX)

## Abbreviations

GDP: gross domestic products
SMOG: Simple Measure of Gobbledygook
