# Supplementary figures and images for: Biomedical and life science articles by female researchers spend longer under review
Source: PLoS Biol. 2026 Jan 20;24(1):e3003574. doi: 10.1371/journal.pbio.3003574 (PMC12818632; doi:10.1371/journal.pbio.3003574)

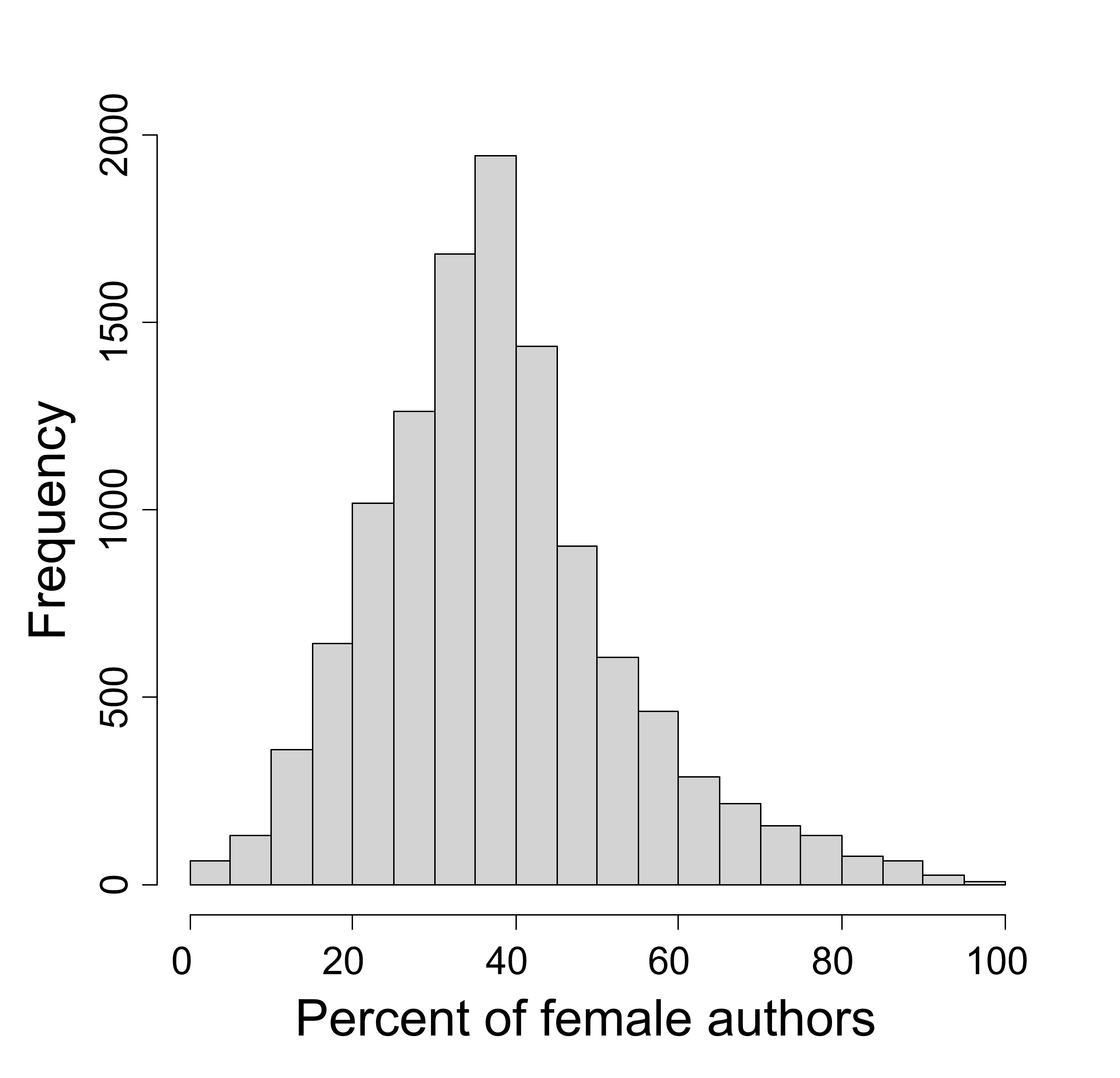

Supplement: S1 Fig — This figure is based on Dataset 1b. The data underlying this Figure can be found in Zenodo (https://doi.org/10.5281/zenodo.17796183). (TIF) [file pbio.3003574.s001.tif]

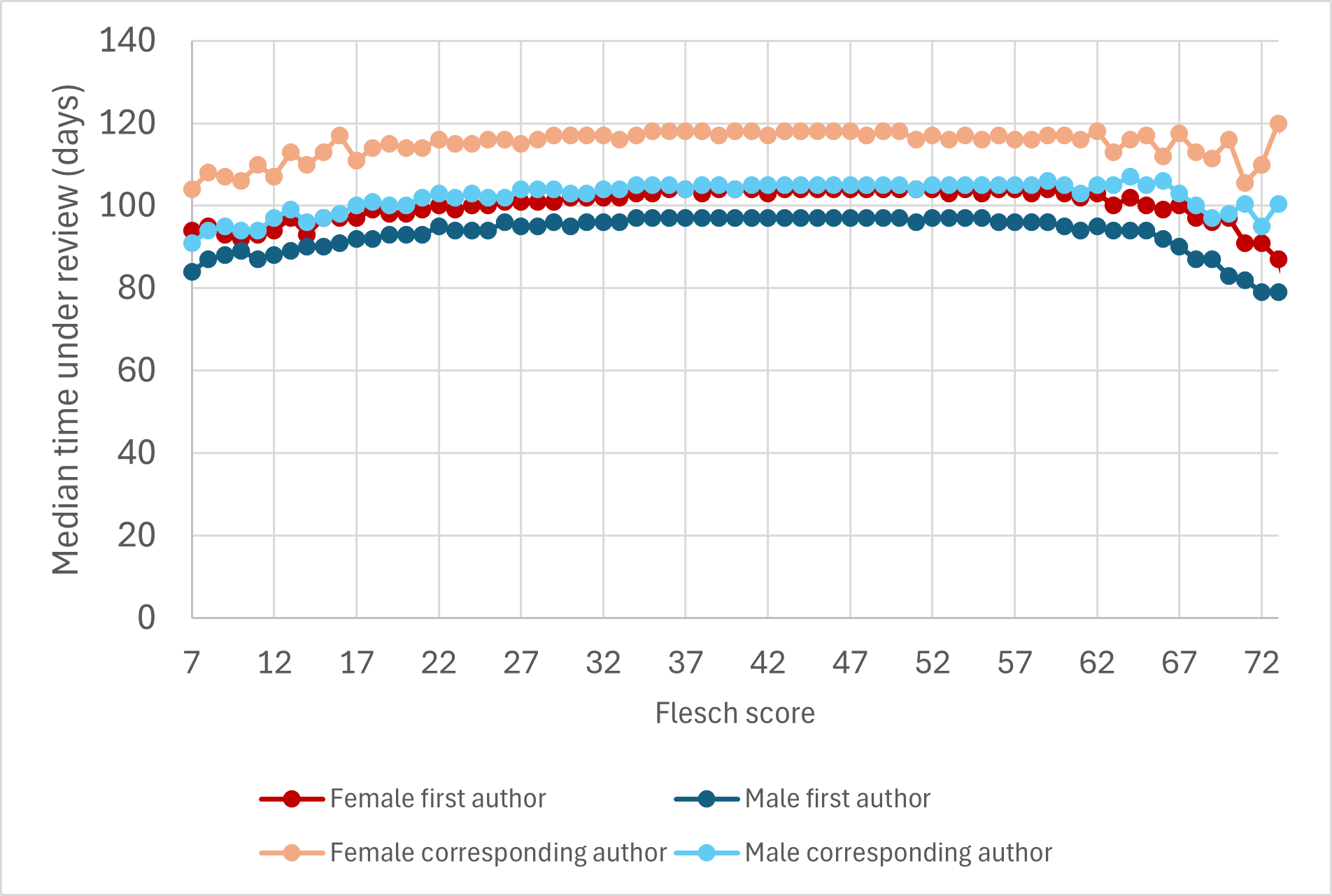

Supplement: S2 Fig — This graph is based on Dataset 2, excluding articles with a missing abstract and those with abstracts’ readability scores outside the range [7, 74). Each category included abstracts with similar scores, down to a unit. Each group is labeled according to its lowest value—e.g., abstracts with a score in the range [10, 11) were included in the “10” category. 99.48% of the articles in the dataset have abstracts with a score in the range [7, 74). The data underlying this Figure can be found in Zenodo (https://doi.org/10.5281/zenodo.17796183). (TIF) [file pbio.3003574.s002.tif]

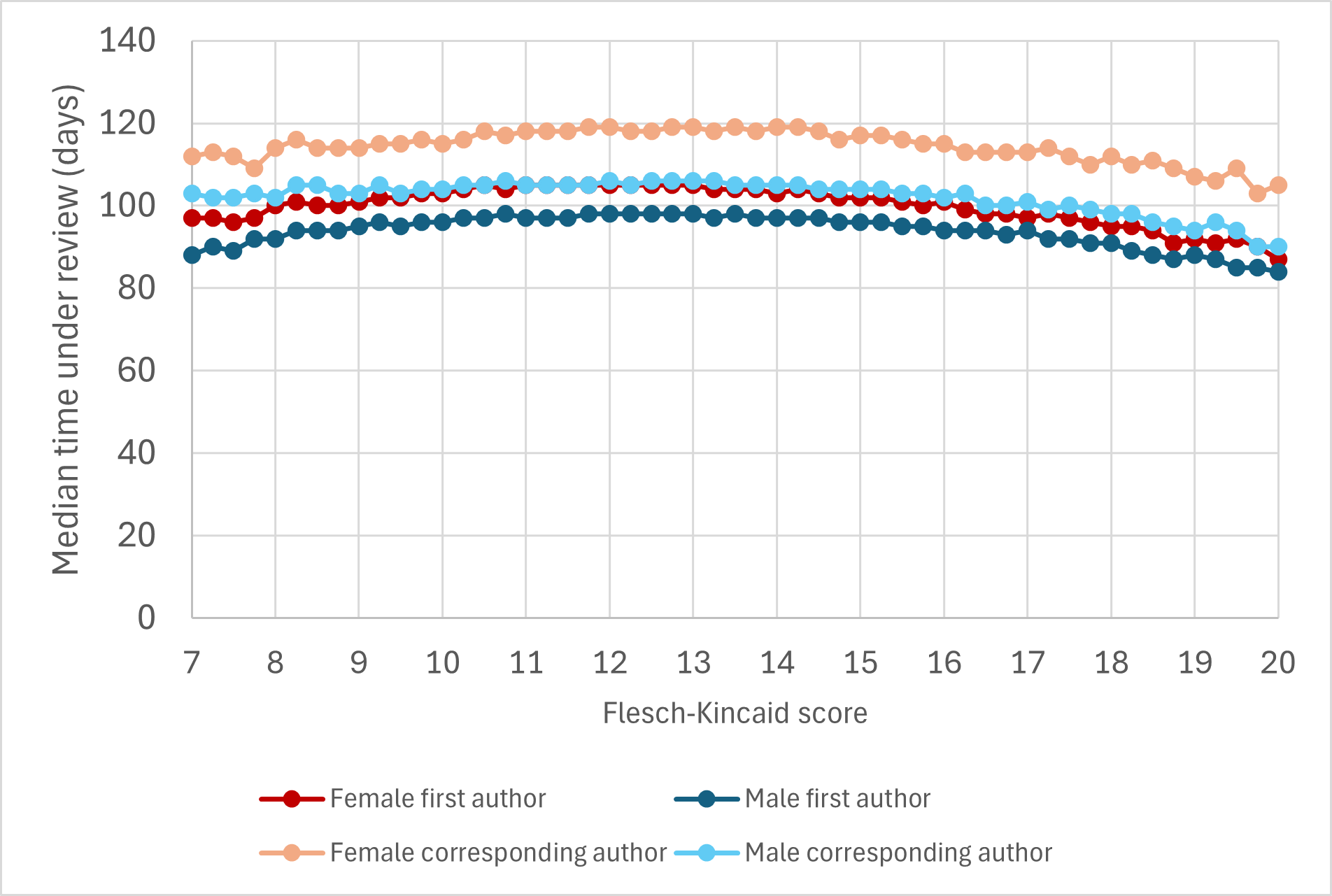

Supplement: S3 Fig — This graph is based on Dataset 2, excluding articles with a missing abstract and those with abstracts’ readability scores outside the range [7, 20.25). Each category included abstracts with similar scores, down to a quarter of a unit. Each group is labeled according to its lowest value—e.g., abstracts with a score in the range [10, 10.25) were included in the “10” category. 98.65% of the articles in the dataset have abstracts with a score in the range [7, 20.25). The data underlying this Figure can be found in Zenodo (https://doi.org/10.5281/zenodo.17796183). (TIF) [file pbio.3003574.s003.tif]

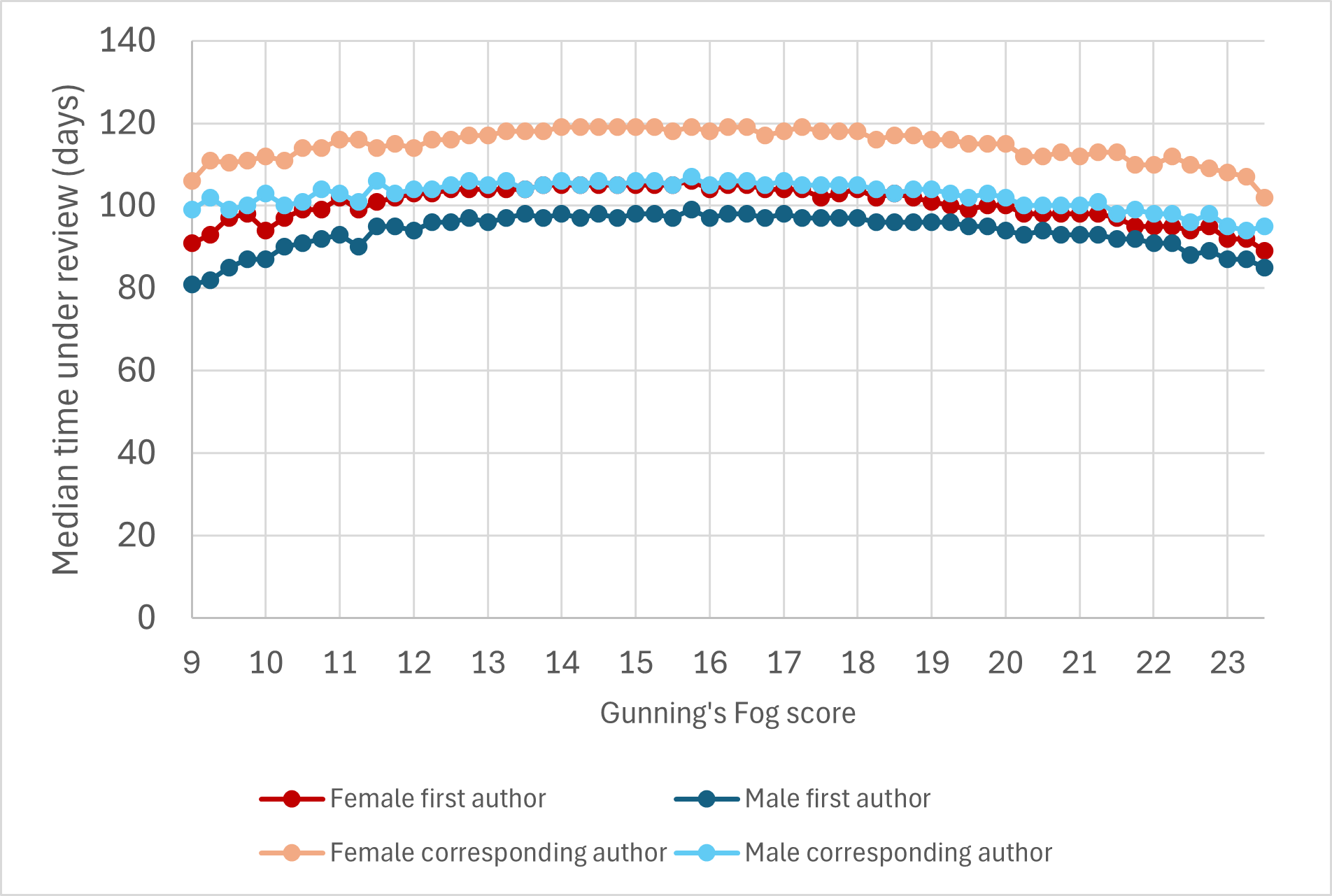

Supplement: S4 Fig — This graph is based on Dataset 2, excluding articles with a missing abstract and those with abstracts’ readability scores outside the range [9, 23.75). Each category included abstracts with similar scores, down to a quarter of a unit. Each group is labeled according to its lowest value—e.g., abstracts with a score in the range [10, 10.25) were included in the “10” category. 98.39% of the articles in the dataset have abstracts with a score in the range [9, 23.75)). The data underlying this Figure can be found in Zenodo (https://doi.org/10.5281/zenodo.17796183). (TIF) [file pbio.3003574.s004.tif]

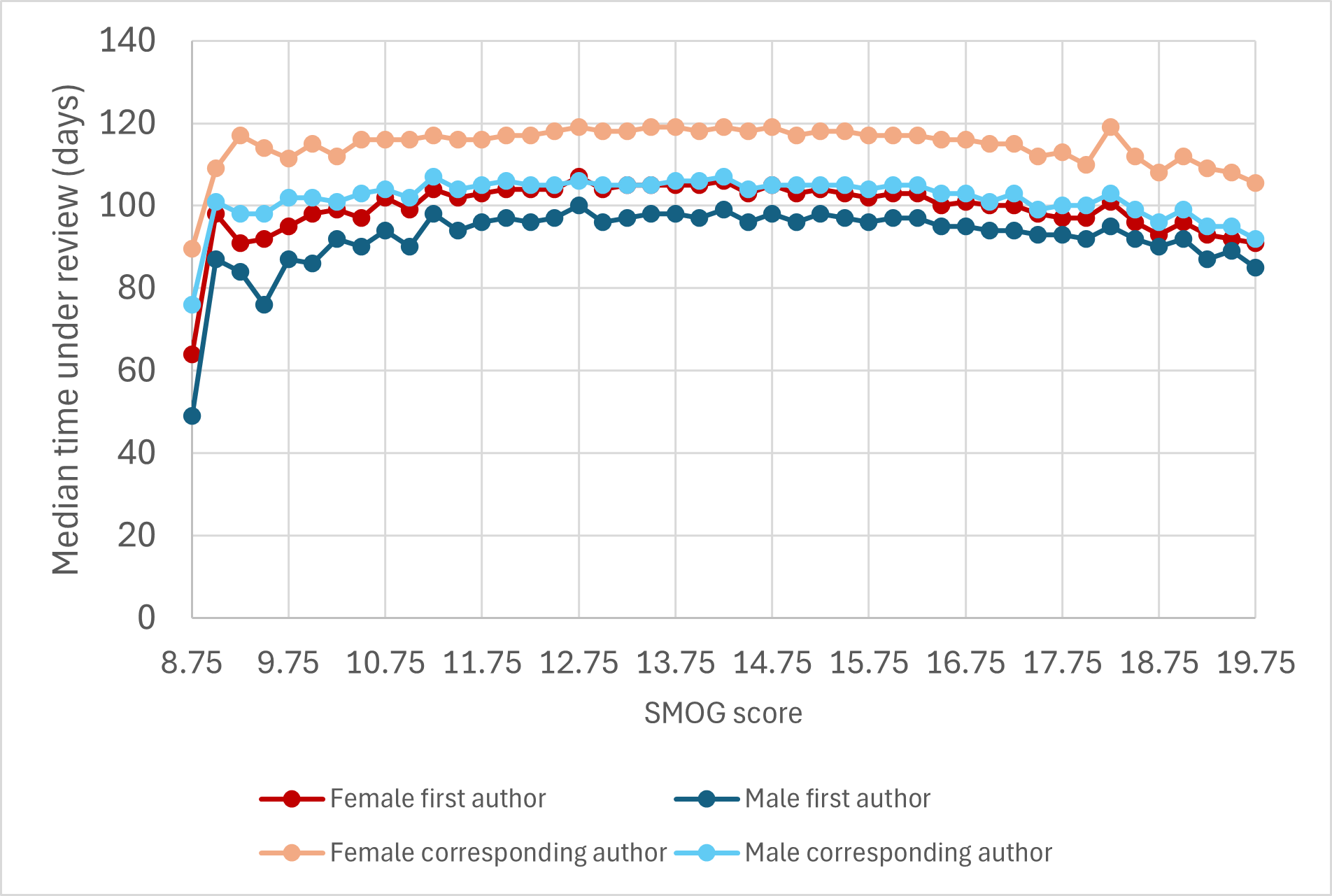

Supplement: S5 Fig — This graph is based on Dataset 2, excluding articles with a missing abstract and those with abstracts’ readability scores outside the range [8.75, 20). Each category included abstracts with similar scores, down to a quarter of a unit. Each group is labeled according to its lowest value—e.g., abstracts with a score in the range [10, 10.25) were included in the “10” category. 98.81% of the articles in the dataset have abstracts with a score in the range [8.75, 20). The data underlying this Figure can be found in Zenodo (https://doi.org/10.5281/zenodo.17796183). (TIF) [file pbio.3003574.s005.tif]
