# Supplementary material for: Biomedical and life science articles by female researchers spend longer under review
Source: PLoS Biol. 2026 Jan 20;24(1):e3003574. doi: 10.1371/journal.pbio.3003574 (PMC12818632; doi:10.1371/journal.pbio.3003574)
Supplement: S1 Text — (PDF) [file pbio.3003574.s006.pdf]

SUPPLEMENTARY RESULTS

Female-authored articles are slightly longer

For each of the 4189 journals with at least 10 articles with a female first author and 10 articles with a male first author, we calculated the median number of pages of articles with female first authors (P_{F1}) and the median number of pages of articles with male first authors (P_{M1}). Across all 4189 journals, the median values of P_{F1}, P_{M1} and P_{F1}/P_{M1} were 8.5 pages, 8 pages and 1, respectively. P_{F1} was higher than P_{M1} in 1343 of the journals, lower than P_{M1} in 489 of the journals, and equal to P_{M1} in 2357 of the journals. The correlation between articles' fraction of female authors and articles' number of pages was positive ($\rho > 0$) in 3100 journals, significantly positive ($\rho > 0$ and $P < 0.05$) in 1264 journals, negative ($\rho < 0$) in 1089 journals, and significantly negative ($\rho < 0$ and $P < 0.05$) in 116 journals.

For each of the 2333 journals with at least 10 articles with a female corresponding author and 10 articles with a male corresponding author, we calculated the median number of pages of articles with female corresponding authors (P_{FC}) and the median number of pages of articles with male corresponding authors (P_{MC}). Across all 2333 journals, the median values of P_{FC}, P_{MC} and P_{FC}/P_{MC} were 9 pages, 8 pages and 1, respectively. P_{FC} was higher than P_{MC} in 689 of the journals, lower than P_{MC} in 300 of the journals, and equal to P_{MC} in 1344 of the journals. The correlation between articles' fraction of female authors and articles' number of pages was positive ($\rho > 0$) in 1791 journals, significantly positive ($\rho > 0$ and $P < 0.05$) in 862 journals, negative ($\rho < 0$) in 542 journals, and significantly negative ($\rho < 0$ and $P < 0.05$) in 67 journals.

Female-authored articles have slightly more co-authors

For each of the 5610 journals with at least 10 articles with a female first author and 10 articles with a male first author, we calculated the median number of authors of articles with female first authors (A_{F1}) and the median number of authors of articles with male first authors (A_{M1}). Across all 5610 journals, the median values of A_{F1}, A_{M1} and A_{F1}/A_{M1} were 5 authors, 5 authors and 1, respectively. A_{F1} was higher than A_{M1} in 1579 of the journals, lower than A_{M1} in 895 of the journals, and equal to A_{M1} in 3136 of the journals. The correlation between articles' fraction of female authors and articles' number of authors was positive ($\rho > 0$) in 4393 journals, significantly positive ($\rho > 0$ and $P < 0.05$) in 2537 journals, negative ($\rho < 0$) in 1217 journals, and significantly negative ($\rho < 0$ and $P < 0.05$) in 338 journals.

For each of the 2901 journals with at least 10 articles with a female corresponding author and 10 articles with a male corresponding author, we calculated the median number of authors of articles with female corresponding authors (A_{FC}) and the median number of authors of articles with male corresponding authors (A_{MC}). Across all 2901 journals, the median values of A_{FC}, A_{MC} and A_{FC}/A_{MC} were 5 authors, 5 authors and 1, respectively. A_{FC} was higher than A_{MC} in 685 of the journals, lower than A_{MC} in 599 of the journals, and equal to A_{MC} in 1617 of the journals. The correlation between articles' fraction of female authors and articles' number of authors was positive ($\rho > 0$) in 2392 journals, significantly positive ($\rho > 0$ and $P < 0.05$) in 1629 journals, negative ($\rho < 0$) in 509 journals, and significantly negative ($\rho < 0$ and $P < 0.05$) in 175 journals.
